# Linking suicide and social determinants of health in South Korea: An investigation of structural determinants

**DOI:** 10.3389/fpubh.2022.1022790

**Published:** 2022-10-25

**Authors:** Yongjun Zhu, Seojin Nam, Lihong Quan, Jihyun Baek, Hongjin Jeon, Buzhou Tang

**Affiliations:** ^1^Department of Library and Information Science, Yonsei University, Seoul, South Korea; ^2^Department of Library and Information Science, Sungkyunkwan University, Seoul, South Korea; ^3^Department of Media and Communication, Sungkyunkwan University, Seoul, South Korea; ^4^Department of Psychiatry, School of Medicine, Sungkyunkwan University, Samsung Medical Center, Seoul, South Korea; ^5^Depression Center, Samsung Medical Center, Seoul, South Korea; ^6^Department of Computer Science, Harbin Institute of Technology (Shenzhen), Shenzhen, China

**Keywords:** social determinants of health (SDOH), suicide, South Korea, Korea Psychological Autopsy Center, structural determinants

## Abstract

**Introduction:**

Studies have shown that suicide is closely related to various social factors. However, due to the restriction in the data scale, our understanding of these social factors is still limited. We propose a conceptual framework for understanding social determinants of suicide at the national level and investigate the relationships between structural determinants (i.e., gender, employment statuses, and occupation) and suicide outcomes (i.e., types of suicide, places of suicide, suicide methods, and warning signs) in South Korea.

**Methods:**

We linked a national-level suicide registry from the Korea Psychological Autopsy Center with the Social Determinants of Health framework proposed by the World Health Organization's Commission on Social Determinants of Health.

**Results:**

First, male and female suicide victims have clear differences in their typical suicide methods (fire vs. drug overdose), primary warning signs (verbal vs. mood), and places of death (suburb vs. home). Second, employees accounted for the largest proportion of murder-suicides (>30%). The proportion of students was much higher for joint suicides than for individual suicides and murder-suicides. Third, among individuals choosing pesticides as their suicide method, over 50% were primary workers. In terms of drug overdoses, professionals and laborers accounted for the largest percentage; the former also constituted the largest proportion in the method of jumping from heights.

**Conclusion:**

A clear connection exists between the investigated structural factors and various suicide outcomes, with gender, social class, and occupation all impacting suicide.

## Introduction

Since the Ottawa Charter for Health Promotion (1) identified prerequisites for health—including peace, shelter, education, food, income, a stable ecosystem, sustainable resources, social justice, and equity—in 1986, a variety of approaches have been proposed to better understand health promotion (2). Particularly, during the past two decades, several studies have highlighted social factors' impact on health (3). Along with medical care, social factors are critical determinants of health [hereafter referred to as social determinants of health (SDOH); act as a critical component of health determinants (4)]. Researchers and organizations have made efforts to present SDOH frameworks that can be used to organize and guide relevant studies. In 2003, the World Health Organization (WHO). Regional Office for Europe published the second edition of *Social Determinants of Health: The Solid Facts* (5) and highlighted 10 SDOH topics including the social gradient, stress, early life, social exclusion, work, unemployment, social support, addiction, food, and transport. In 2008, the Centers for Disease Control and Prevention in the U.S. Department of Health and Human Services released a report and discussed eight SDOH that are closely related to health equity, which include access to care, insurance coverage, employment, education, access to resources, income, housing, and transportation (6).

One of the most comprehensive SDOH frameworks was introduced a decade ago. In 2010, WHO's Commission on Social Determinants of Health (CSDH) proposed a conceptual framework (7) for studying SDOH by integrating three elements: social-economic and political context, structural determinants, and intermediary determinants. Structural determinants “generate or reinforce social stratification in the society and that define individual socioeconomic position” (7) (p. 30) and are measured by proxies such as income, education, occupation, social class, gender, and race or ethnicity. Structural determinants operate through intermediary determinants that include three categories: material circumstances (including physical environment, consumption potential, and physical working and neighborhood environments), psychosocial circumstances (including psychosocial stressors, stressful living circumstances and relationships, and social support and coping styles), and behavioral and biological factors (including smoking, diet, alcohol consumption, and lack of physical exercise). Later, Braveman et al. (8) introduced neighborhood conditions, working conditions, education, income and wealth, race and racism, and stress as the upstream SDOH that play more fundamental roles in health promotion and shape downstream SDOH such as health-related knowledge, attitudes, beliefs, and behaviors. The aforementioned approaches to SDOH differ in the level of granularity, overlap to a certain extent, and complement each other. Although there is no unified system encompassing every concept listed above, each of them has broadened our understanding of SDOH and provided a valid framework in which to study various healthcare problems.

Suicide is also a global problem, as more than as 700,000 people die by suicide every year (9). Suicide is the 12^th^ leading cause of death in the U.S., with 45,979 Americans (13.48 per 100,000 individuals) died by suicide and another 1.2 million attempting suicide in 2020 (10). In the same year, 13,195 South Korean (25.7 per 100,000 individuals) died by suicide, making it as the fifth leading cause in the country (11). Suicide is closely related to various social factors, and successful suicide prevention programs have attempted to address problems such as health inequality (12). Social determinants of health were also reported as strong predictors of suicide risk (13, 14). Nevertheless, we have limited understanding about the social determinants of suicide for two main reasons. First, there has been no attempt to broadly investigate suicide under a comprehensive SDOH framework. Second, detailed national-level suicide data that include various social factors are not easily accessible. To address this research gap, the Korea Psychological Autopsy Center with the support of the Ministry of Health and Welfare of South Korea has been curating data on all 67,331 individuals who died by suicide between 2013 and 2017 (15). This is the most recent, detailed, and complete data for understanding various social determents of suicide in South Korea. In this study, we aim to systematically link suicide in South Korea with the SDOH conceptual framework proposed by the CSDH by mapping variables used to describe individuals' socioeconomic characteristics onto SDOH concepts. To our knowledge, this is the first study to link national-level suicide registry data with the CSDH framework and explore social determinants of suicide using population-level data.

## Literature review

Social determinants of suicide have been studied in two main ways: at the ecological level and at the individual level. Ecological-level studies use publicly available data provided by governmental institutions and study the relationships between suicide rates and various socioeconomic measures such as the Gini index, unemployment rate, and urbanization (16–20). Because the units of analysis of ecological-level studies are countries, regions, and groups of people, only variables available at the ecological level have been studied. Therefore, the analyses were performed at the macro level with a focus on common social contexts, such as understanding how suicide rates of regions differ under different social and economic conditions. On the other hand, individual-level studies facilitate deeper understanding by utilizing a wide range of individual variables such as biological markers, diet, medical and psychiatric diagnoses, and behavioral patterns.

Although such investigations have not been comprehensive or exhaustive, researchers in various countries have examined social determinants of suicide at the individual level. Earlier studies investigated social determinants of suicidal ideation, suicide attempts, and related behaviors *via* self-reported data. Studies conducted in the U.S. have focused on specific groups including high school students, college students, nurses, local community members, and veterans. Bonner and Rich (21) surveyed 158 U.S. college students and reported that loneliness, irrational beliefs, and weak adaptive reasons for living are associated with suicidal behavior. Further, in a sample of 13,639 U.S. high school students, Swahn and Bossarte (22) found preteen alcohol use initiation to be significantly associated with suicidal ideation and suicide attempts. Tsai et al. (23) surveyed 72,607 nurses (ages 46 to 71), finding that social integration has an inverse association with suicide and that women with higher social integration had a more than three-fold lower risk for suicide. In another survey (*N* = 528) investigating social determinants of suicide in Monroe County Florida, the researchers found that compared to those not at risk, those at risk for suicide exhibited more depression, poorer mental health, and more activity limitations due to their health (24). Among the determinants measured, housing instability was the most influential, and renting a home was associated with a more than three-fold increase in suicide risk. Lastly, Blosnich et al. (13) investigated social determinants of suicidal ideation and attempts among U.S. veterans (*N* = 293,872). The study showed that non-specific psychosocial needs were the most prevalent social risk factor among the study participants, followed by housing instability and employment or financial problems.

Although not as active as those in the U.S., researchers in countries such as China, Ghana, Australia, South Africa, and Kenya have investigated the relationships between SDOH and suicide. Zhang and Jin (25) investigated determinants of suicidal ideation in a survey of 320 Chinese and 452 American college students, showing that these social determinants differ across demographic groups. They found that among American students, family cohesion and religiosity are negatively related to suicidal ideation, whereas there was a positive correlation between religiosity and suicidal ideation for Chinese students. Another survey (*N* = 375) compared determinants of suicidal ideation between American and Ghanaian college students, with family cohesion (but not religiosity) being revealed as a significant predictor (26). Gender was a significant determinant among Ghanaians, as Ghanaian women reported higher suicidal ideation compared to men. In a survey in Australia (*N* = 10,641), the researchers found that low levels of education and occupational status are positively associated with suicide attempts (27). In another Australian study (*N* = 8,463) revealed marital status, employment status, perceived financial adversity, and mental health service use as important determinants of suicidal ideation and attempts (28). Shilubane et al. (29) surveyed and interviewed 14 black South African adolescents to explore factors related to suicide attempts. Significant determinants included lack of knowledge about available counselors, conflicts in interpersonal relationships, perceived accusations of negative behavior, inadequate social support, past family and peer suicide attempts, and poor living circumstances. In a study of 532 young Kenyan men (18–34 years old), the researchers treated lower collective self-esteem, hopelessness, less meaning in life, and more loneliness as mediator variables, finding that suicidal ideation was significantly higher among those who reported lower subjective social status (30).

In the aforementioned studies based on self-reported data, researchers used proxy variables (e.g., suicidal ideation) to understand suicide. Given that most individuals reporting suicidal ideation do not die by suicide (31), these studies' results may not be a completely accurate reflection of the social determinants of death by suicide. Therefore, it is possible that we may have a partial understanding of SDOH's relationship with suicide. A nationwide system compiling data on completed suicides is thus critical for more accurately understanding the social determinants of suicide.

## Materials and methods

### The national suicide data in South Korea

South Korea's Ministry of Health and Welfare established the Korea Psychological Autopsy Center in 2014 to curate all cases of completed suicide between 2013 and 2017 in collaboration with 254 police stations and 32 trained investigators (32). According to Statistics Korea (15), 67,331 individuals died by suicide during the 5-year period. In this ongoing national project, investigators manually review each case to ensure data quality and accuracy. At the time of writing, 23,668 cases in eight major cities and provinces including Seoul, Daejeon, Gwangju, Jeju, Sejong, Chungcheongnam-do, Chungcheongbuk-do, and Gangwon-do had been documented. More than 30 individual-level variables grouped into four categories have been collected, as shown in Table 1. This is the most recent, detailed, and complete data for understanding various social determinants of suicide in South Korea, and we adopted the CSDH conceptual framework in analyzing these data.

**Table 1:** List of individual-level variables appearing in South Korean national suicide data.

**Category**	**Variables**
Socio-demographic	Gender, age, residential address, housing type, housing tenure, level of education, employment status, occupation, marital status, co-living status
Suicide-related	Date/estimated time of death, date/time of finding, place of death, suicide method, existence of joint suicide victim, existence of murder-suicide victim, past self-harm, past suicide attempts, first finder, existence of suicide note
Suicide cause-related	Workplace issues, financial issues, family relationships, interpersonal relationships, physical disorders, physical disabilities, psychiatric symptoms, diagnosed psychiatric disorders, psychiatric treatment history, alcohol consumption at time of death
Observed by informant	Relationship with the victim, changes before death as warning signs (verbal, behavioral, mood)

### The CSDH conceptual framework

The CSDH conceptual framework (7) integrates three elements: social-economic and political context, structural determinants, and intermediary determinants. Social-economic and political context is a broad concept that refers to factors in society that cannot be measured at the individual level, which include governance, macroeconomic policies, social policies, public policies, and cultural and societal values. Structural determinants refer to the interplay between socioeconomic-political context and individuals' resultant socioeconomic position. Socioeconomic position is operationalized with six major variables: income, education, occupation, social class, gender, and ethnicity. Structural determinants operate through intermediary determinants that include three categories: material circumstances (including physical environment, consumption potential, and physical working and neighborhood environments), psychosocial circumstances (including psychosocial stressors, stressful living circumstances and relationships, and social support and coping styles), and behavioral and biological factors (including smoking, diet, and lack of physical exercise).

### Integrating the national suicide data and the CSDH conceptual framework

**Figure 1:**
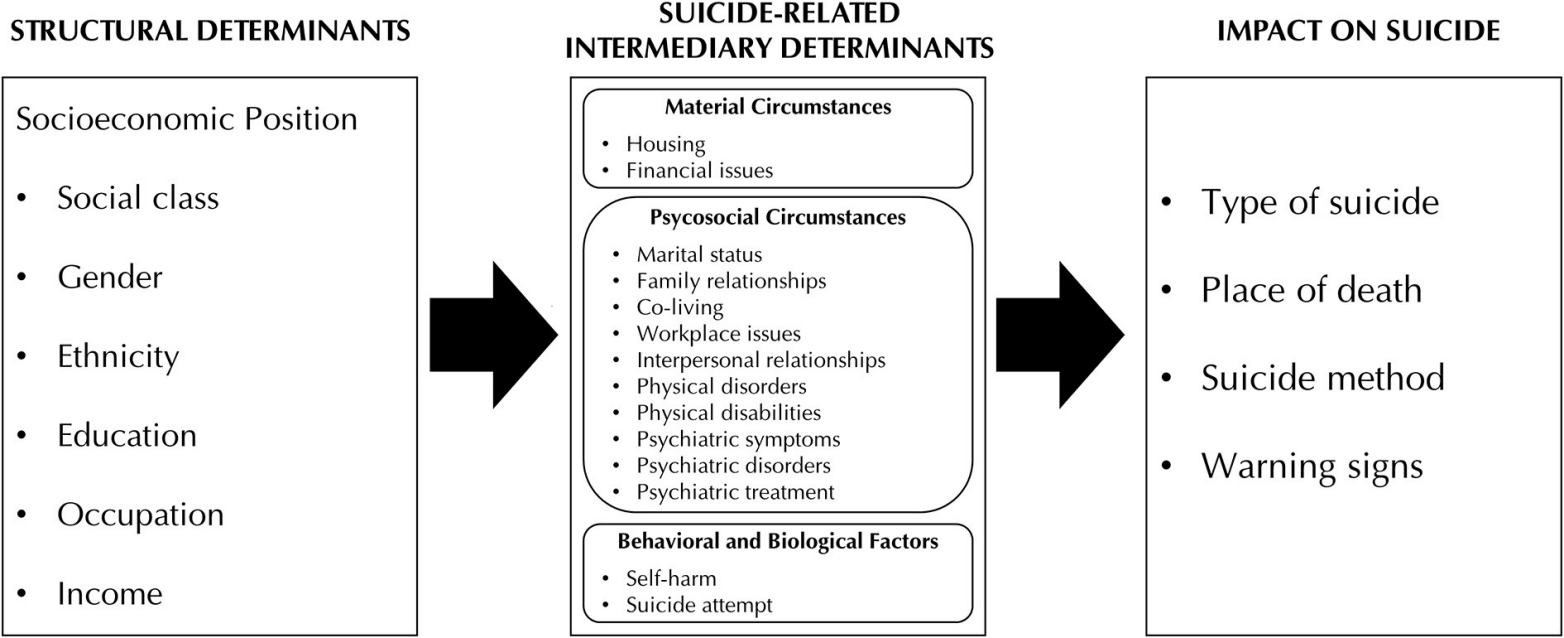
Conceptual framework for understanding social determinants of suicide.

Based on the abovementioned concepts, we adapted the CSDH framework for use with our data. Figure 1 shows the conceptual framework for understanding social determinants of suicide. Based on the original CSDH framework, we included the six major variables capturing socioeconomic position. Among which, for this study, we focused on three variables that were available from the national suicide data: gender, social class, and occupation. It is a limitation that only partial social determinants were explored in the study. All categories under each variable were reference from the codebook for data users provided by the Korea Psychological Autopsy Center. Gender consisted of two categories: male and female. Social class was derived from suicide victims' employment status, which comprised seven categories: employee, self-employed, unemployed, student, housemaker, military or social service worker, and other economically inactive individuals. “Other economically inactive” referred to those who are not working because they do not have the ability or intention to work (an individual variable), including the elderly, individuals with disabilities, and those preparing for state exams; however, students, housemakers, and military or social service workers had their own categories (11) (p. 71). Meanwhile, occupations encompassed 11 categories with 10 major occupational clusters: manager, professional, office worker, service worker, salesperson, primary industries worker, technician, mechanic, laborer, and soldier. “Technicians” are individuals with jobs that place more importance on the skill or craft, while “mechanics” are individuals with more machine-oriented jobs, such as operating and assembling machinery (33) (pp. 19–23). The last category was “illegal business-related workers,” which refers to those involved in illegal businesses such as prostitution or gambling.

Following the structural determinants, we grouped the suicide-related intermediary determinants (i.e., factors that directly impact suicide) into three categories. Psychosocial circumstances were the focus of our investigation, although we also explored material circumstances and behavioral factors. As for the outcomes, we investigated how structural and intermediary determinants affect the type of suicide, place of death, suicide method, and warning signs for suicide.

**Figure 2:**
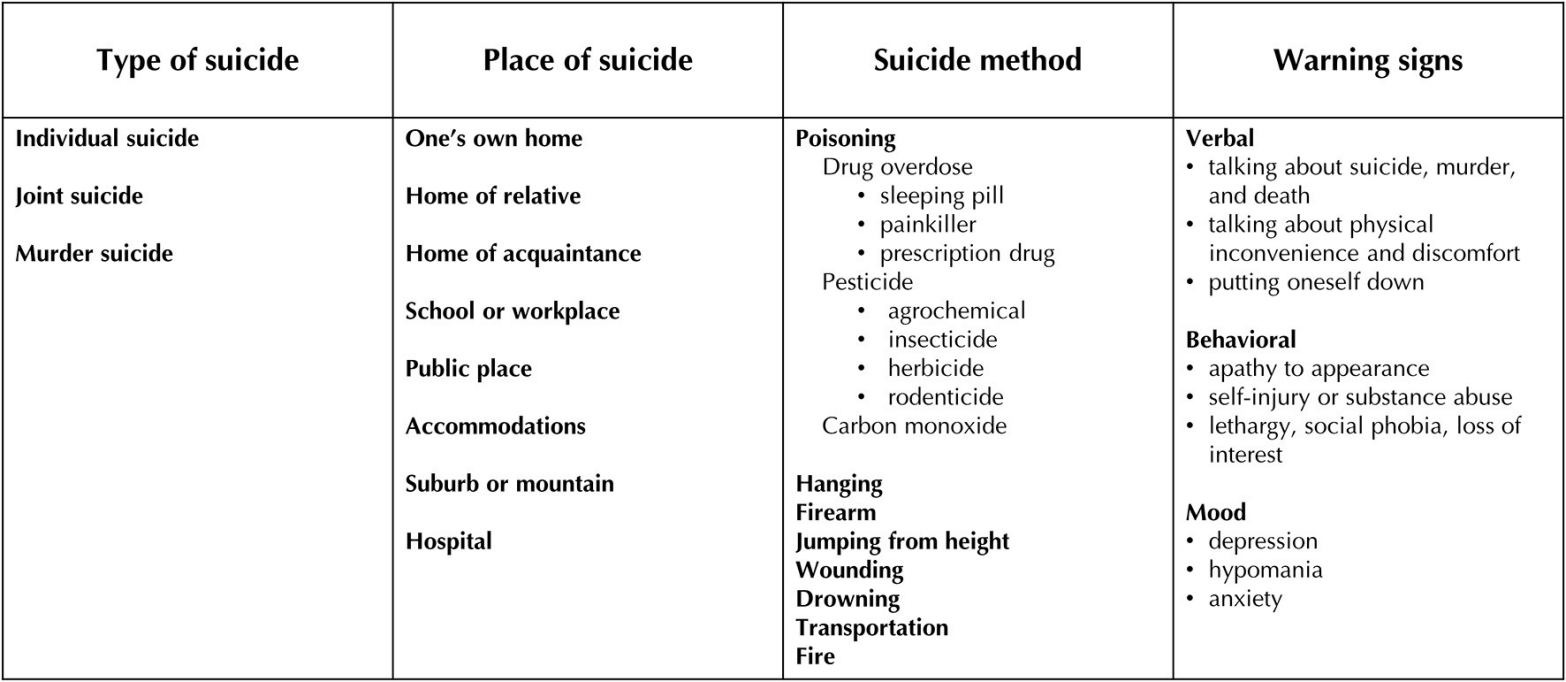
Four categories of suicide outcomes.

Figure 2 provides details about these outcomes. Type of suicide indicates a suicide's correspondence to one of three categories: individual suicide, joint suicide, or murder-suicide. We also assumed that social factors can impact the place in which someone chooses to die by suicide. We investigated suicide methods in relation to structural and intermediary determinants. Finally, one or more warning signs of suicide were collected by interviewing individuals who knew the victims. Warning signs were grouped into three categories: verbal, behavioral, and mood.

## Results

In the rest of the paper, we report our findings concerning the four outcomes in relation to three structural determinants: gender, social class, and occupation.

### Types of suicide and social determinants

Across gender and all social classes and occupations, individual suicide was the most prevalent suicide type followed by joint and murder-suicide. For all suicide types, the number of male victims exceeded that of female victims. Compared with the proportion of men committing individual suicide (70%), the proportion of men was lower for joint suicide (56%) and higher for murder-suicide (79%). A closer look at the victims revealed that joint suicide for both genders were most often completed with strangers they met for the purpose of dying (100 cases for male and 45 cases for female), followed by a spouse (40 cases). Meanwhile, murder-suicide for male mostly involved a spouse or lover (36 cases total) and a child for female (11 cases).

**Figure 3:**
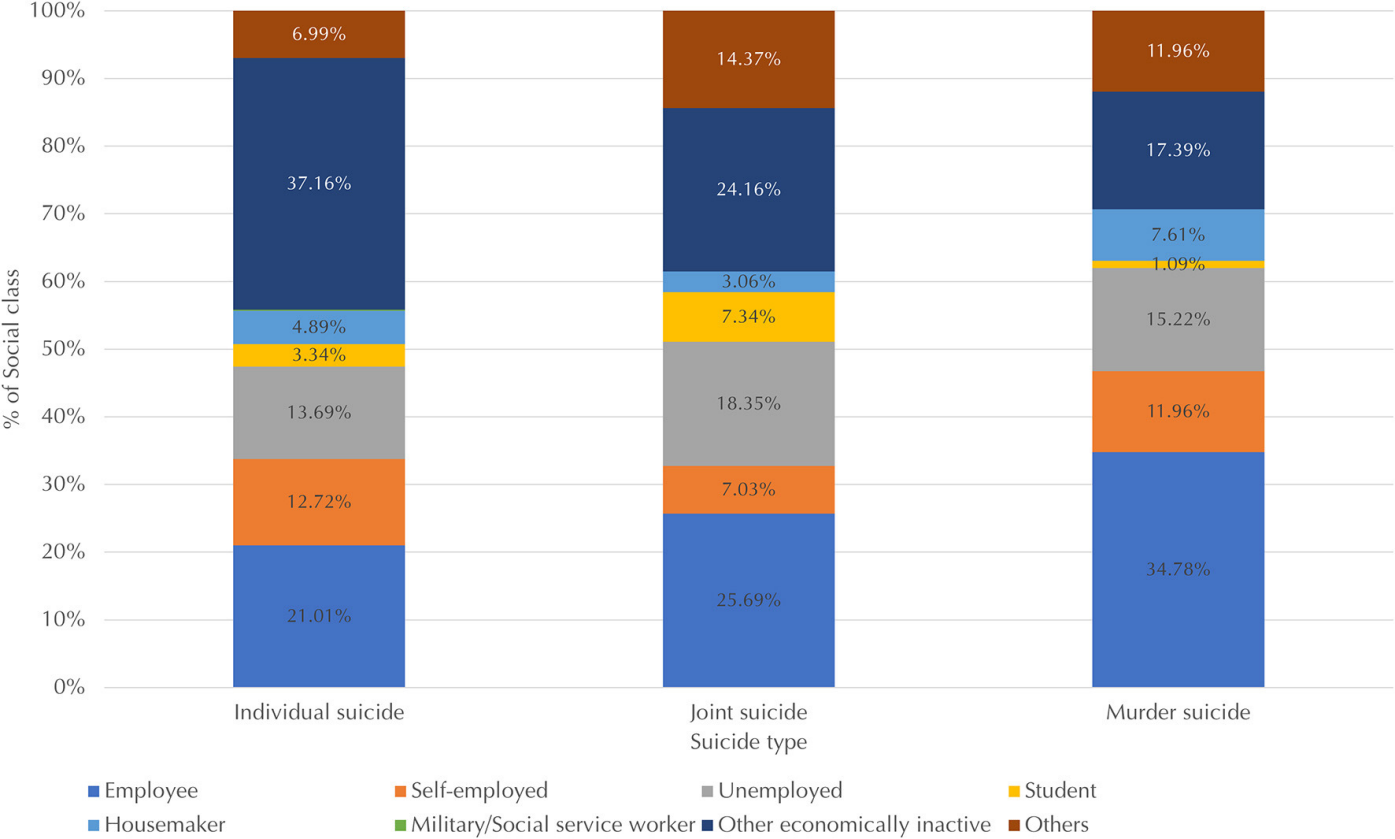
Distribution of social classes (measured by employment status) across suicide types. Data for military or social service workers, which accounted for < 1% of victims for each suicide type, are not shown due to limited space.

Figure 3 shows the distribution of social classes across suicide types. Other economically inactive and unemployed individuals together accounted for 51% of individual suicides, 43% of joint suicides, and 33% of murder-suicides. The percentages of employees and housemakers, who are economically dependent on others (i.e., an employer or a breadwinner), were higher for murder-suicide (35 and 7.6%, respectively) than for other suicide types. Meanwhile, students and unemployed individuals had higher percentages for the joint suicide type (7.3 and 18%, respectively) compared to other types of suicide. The two groups are generally exposed to enduringly and consistently stressful circumstances, such as stress from their studies (34) (p. 32), careers, and interpersonal relationships. In terms of individual suicide, other economically inactive individuals comprised the largest proportion and constituted a higher percentage (37%) compared to those for other suicide types (24% and lower).

With respect to individual suicides, victims' occupations were relatively evenly distributed (except soldiers), though laborers and salespeople were the most common individual suicide victims. For joint suicides, individuals working in the service industry or in illegal businesses accounted for higher percentages (19 and 16%, respectively) than for other suicide types. These workers share the characteristic of economic instability, supported by the fact that service workers on average earn the lowest monthly wages in South Korea (35). On the other hand, for murder-suicides, mechanics and laborers accounted for higher percentages (14 and 20%, respectively) than for other suicide types. Both occupations, generally known as blue-collar workers, consist in manual labor that requires long working hours and is highly physically demanding, supported by the fact that mechanics on average have the longest working hours of all occupations in South Korea (35). These two occupations are also both male-dominated and characterized by low educational attainment, as they have the largest proportion of workers with a high school education or less (35).

### Places of death and social determinants

Across gender and most social classes (except military or social service workers) and occupations (except *soldier*), people died most frequently in one's own home, followed by public places. For all places of death, the number of male suicide victims exceeded that of female victims. Compared with the proportions of men who died by suicide in other places (ranging from 74% to 77%), the proportions of men who died by suicide in suburban or mountainous areas (93%) and at school or in the workplace (88%) were higher, but they were lower than the proportions of men who died by suicide in one's own (63%), a relative's (60%), or an acquaintance's home (56%).

**Figure 4:**
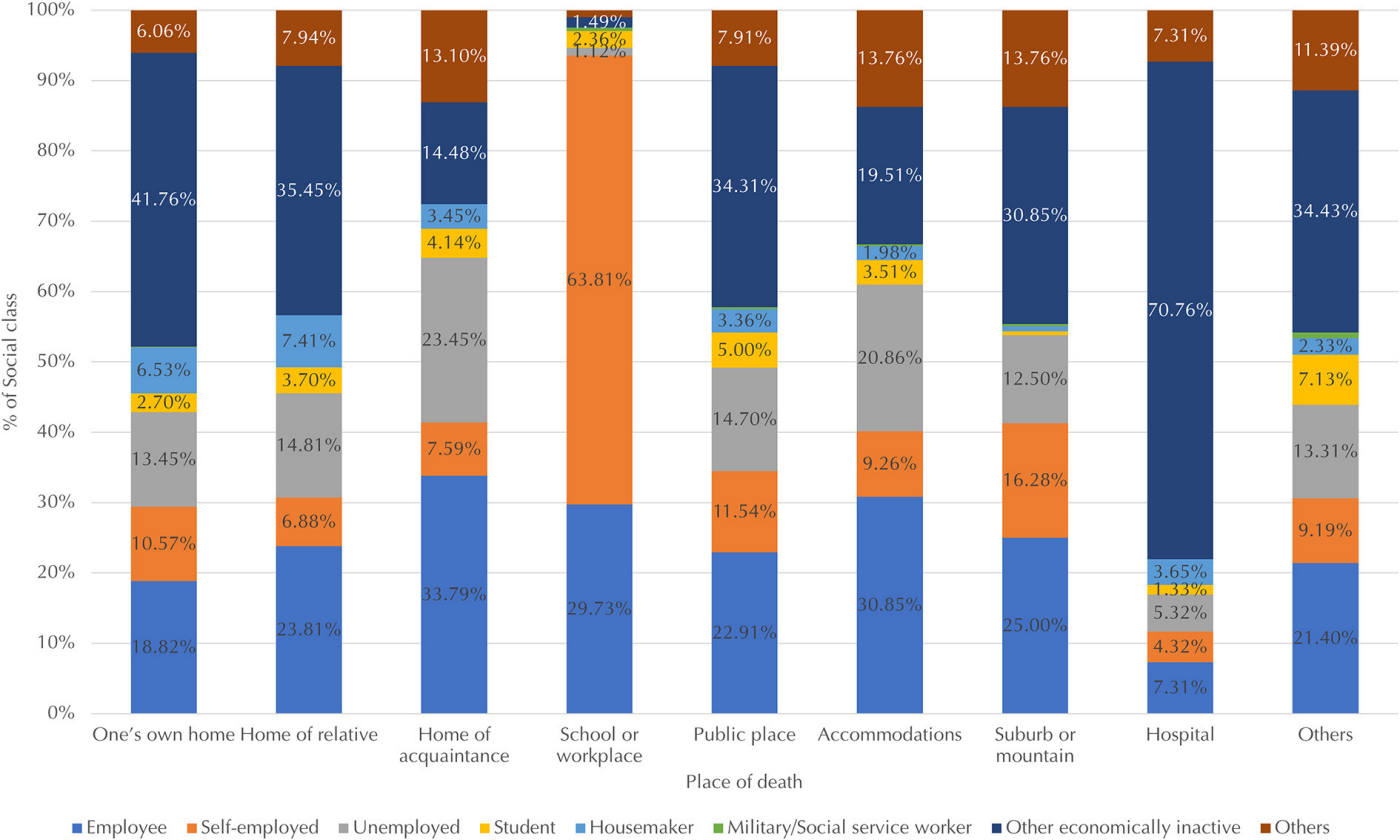
Distribution of social classes (measured by employment status) across places of death. Data for social classes that accounted for < 1% of victims for place of death, including military or social service workers, are not shown due to limited space.

Figure 4 shows the distribution of social classes across places of death. For most places of death, the largest number of suicide victims were from the other economically inactive group. Some exceptions included school or workplace suicides, for which self-employed individuals accounted for the majority (64%), followed by employees (30%). Employees died by suicide at higher rates in school or the workplace, in an acquaintance's home, and in accommodations compared with other places (30% and higher vs. 25% and lower). For suicides in one's own home or a relative's home, housemakers had higher percentages than they did for other places (over 6.5% vs. under 3.7%). For suicides in hospitals, the other economically inactive group, which includes the elderly and individuals with disabilities, had higher percentages than they did in other places (71% vs. 42% and lower). These findings largely indicate that individuals are likely to die by suicide in places where they spend most of their time.

Regarding occupation, the percentages of managers and salespeople who died by suicide in school or the workplace were higher than for other places (both 21% vs. under 12% and 17%). Those working in primary industries or in illegal businesses died by suicide at higher rates in hospitals (19%) than in other places (under 13% and 11%).

### Suicide methods and social determinants

Across gender, social class, and occupation, the top five suicide methods were hanging, pesticides, carbon monoxide, jumping from heights, and drowning. Specifically, except for students and soldiers, hanging was the most frequently used method.

For all suicide methods, the number of male suicide victims exceeded that of female victims. Compared with the proportion of men using the methods of pesticides, hanging, drowning, wounding, and carbon monoxide (67, 71, 75, 78, and 79%, respectively), the proportions of men who died by drug overdose (52%) and jumping from heights (56%) were lower, while the proportions were higher for suicides using fire (88%) and transportation (81%). Meanwhile, the few who died by firearm were men. These findings show that women preferred less lethal methods than did men.

Regarding social class, excluding the few who died by firearm, for nearly all suicide methods, other economically inactive individuals and employees represented the largest proportion of suicide victims. Some exceptions included pesticides, for which other economically inactive individuals accounted for the majority (56%), followed by self-employed individuals (18%), both of which had higher percentages than they did for other methods. Additionally, the percentages of employees who died by fire and carbon monoxide poisoning were higher than for other methods (over 28% vs. under 23%). Meanwhile, the percentages of students who died by jumping from heights and from drowning were higher than those who died by other methods (8.9% and higher vs. 3.0% and lower).

**Figure 5:**
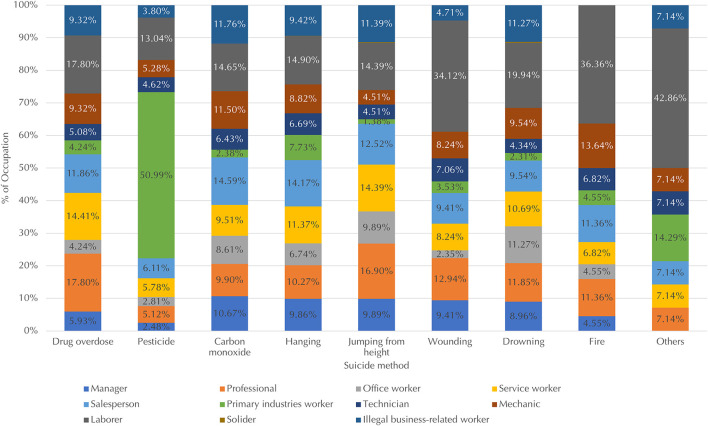
Distribution of occupations across suicide methods, excluding firearm and transportation, which accounted for approximately 0.1% of the total suicide victims. Data for occupations that accounted for < 1% of victims for each suicide method, including soldiers, are not shown due to limited space.

Figure 5 shows the distribution of occupations across suicide methods. Excluding the few who died by firearm (*n* = 5) and transportation (*n* = 11), laborers accounted for the most victims of most suicide methods. Some exceptions included pesticides, most frequently used by primary industries workers, with a percentage (51%) higher than that for other methods. For suicides by fire, mechanics and laborers had higher percentages (14 and 36%, respectively) than for other methods. Laborers accounted for a high percentage (34%) of wound-induced suicides, as well. This indicates that individuals in these occupations preferred lethal methods more commonly than did those in other occupations. Both groups are blue-collar workers sharing several common characteristics, including performing primarily manual labor, which enables access to certain tools that could be used in suicide, and being in a male-dominated profession (35). On the other hand, professionals (generally known as white-collar workers) had higher percentages (over 17% vs. under 13%) of suicide by drug overdose and jumping from heights, indicating their preference for less lethal methods. Compared to other occupations, these jobs require a higher level of education, and these workers on average earn the second highest monthly wages in South Korea (33, 35) (p. 14). The percentages of workers in the service industry, which is largely female-dominated (35), were also higher for the abovementioned two less lethal suicide methods compared to other methods (both 14% vs. under 11%).

### Warning signs of suicide and social determinants

Regarding gender, male suicide victims frequently showed verbal, mood-related, and behavioral warning signs in descending order (71, 65, and 53%, respectively). On the other hand, female victims exhibited mood-related signs the most, followed by verbal and behavioral signs (79, 77, and 55%, respectively). Specifically, most female victims displayed signs of depression, with a much higher percentage of depressive warning signs compared to men (76% vs. 62%).

**Figure 6:**
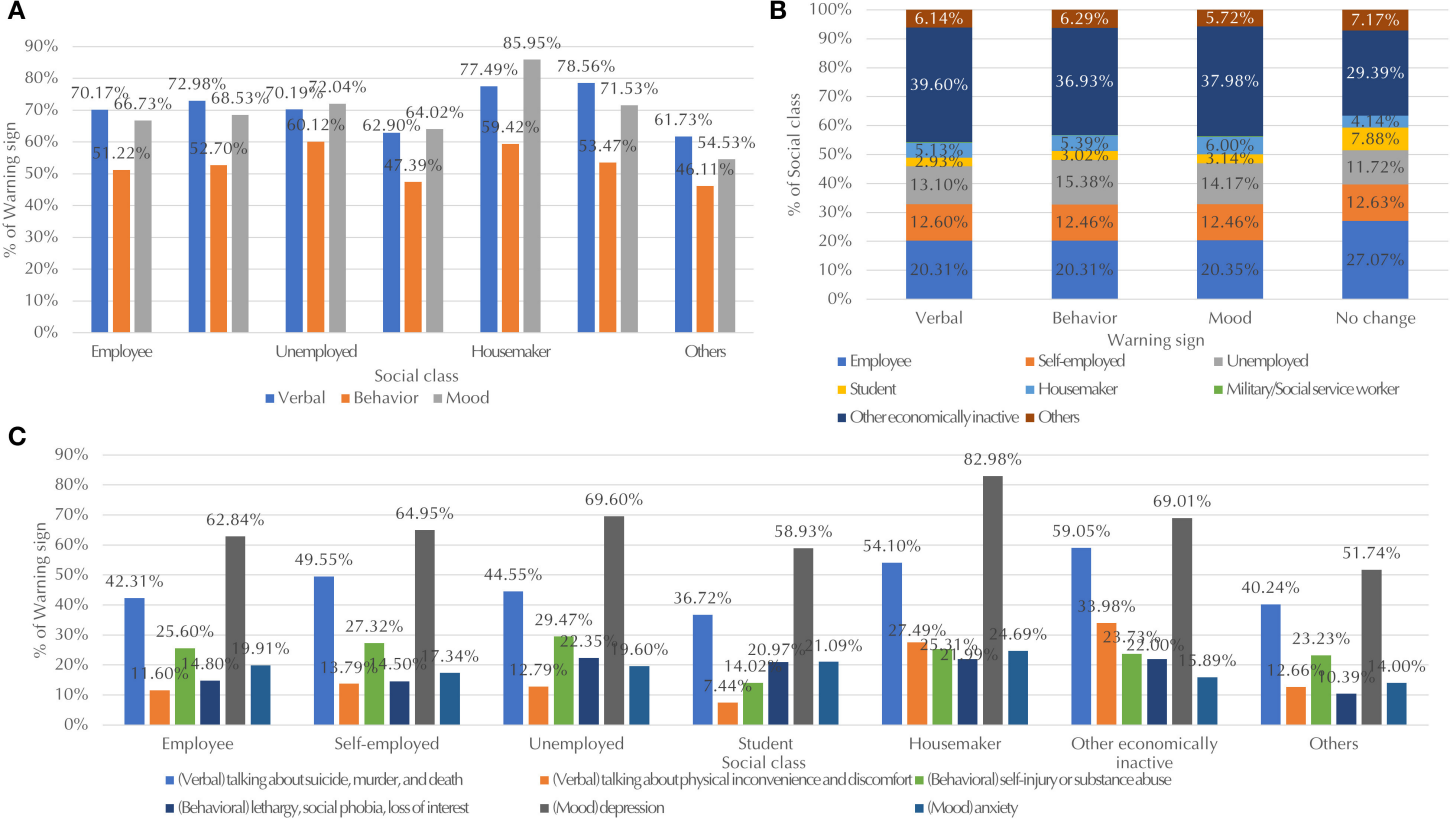
**(A)** Distribution of warning signs across social classes (measured by employment status), **(B)** distribution of social classes across warning signs, and **(C)** distribution of subcategories of warning signs across social classes. For **(A,C)**, military or social service workers, which accounted for approximately 0.2% of the total suicide victims, were excluded. Additionally, for **(C)**, only subcategories of warning signs that differed across social classes are shown, and subcategories that accounted for < 10% of the total suicide victims were excluded. In **(B)**, data for military or social service workers, which accounted for < 1% of victims displaying each warning sign, are not shown due to limited space.

Regarding social class, as shown in Figure 6A, unemployed individuals, students, and housemakers showed mood-related, verbal, and behavioral warning signs in descending order, while in other social classes, the most frequent warning sign was verbal. For housemakers, as shown in Figure 6C, the percentage of mood changes, specifically signs of depression, was much higher than those in the other classes (83% vs. under 70%). Additionally, for housemakers and other economically inactive individuals, the percentages of verbal warning signs were higher than they were in other classes (77% and higher vs. 73% and lower). Specifically, among the verbal warning signs, the percentages of those mentioning physical inconvenience and discomfort (27% and higher vs. 14% and lower) as well as suicide, murder, and death (54% and higher vs. 50% and lower) were higher in these two classes compared to other classes. Although the difference was relatively small, the behavioral signs of self-injury or substance abuse were observed at higher rates among unemployed individuals than among those of other classes (29.5% vs. under 27.3%). As shown in Figure 6B, higher percentages of students and employees showed no warning signs (7.9 and 27%, respectively) compared to showing any of the three warning signs. As both groups study or work in organizations with many colleagues, this shows that they often behave discreetly with respect to their suicidal ideation. On the other hand, the economically inactive group had a lower percentage of individuals not showing warning signs compared to those showing any signs (29% vs. over 37%).

For all occupations except professionals, for whom mood-related warning signs were most common, changes were frequently observed in the form of verbal signs, followed by mood-related and behavioral signs. Particularly, verbal signs of mentioning suicide, murder, and death as well as physical inconvenience and discomfort accounted for higher percentages in primary industries workers (53 and 22%, respectively) than in other occupations. Additionally, though the differences were relatively small, the percentages of self-injury or substance abuse, which are behavioral signs, were higher among technicians and laborers than among those in other occupations (over 31.4% vs. under 28.8%). Both professions are male-dominated (35), with 89% of the laborers who died by suicide being male Meanwhile, individuals involved in illegal businesses, who are economically unstable and work without legal protection, showed a higher percentage of the mood-related sign hypomania than did those in other occupations (5.5% vs. under 3.3%).

## Discussion

In this study, we investigated the impact of three structural factors—gender, social class (as measured by employment status), and occupation—on four suicide-related outcomes—types of suicide, places of suicide, suicide methods, and warning signs—by integrating data from the national suicide registry in South Korea into the WHO's CSDH framework. These data included 23,668 suicide cases between 2013 and 2017 in eight major cities and provinces of South Korea compiled by the Korea Psychological Autopsy Center in collaboration with 254 police stations and 32 trained investigators. Several findings are worthy of further discussion.

### Gender and suicide

The number of male suicide victims was greater than that of female victims across all suicide types, all places of death, and all suicide methods. The proportion of male suicide victims was higher in cases of murder-suicide than in cases of individual and joint suicide, whereas the proportion of female suicide victims was higher for joint suicides than for the other two suicide types. Several studies have consistently identified male as the common perpetrator of murder-suicides mostly involving spouse or lover and have tried to describe the reason behind these phenomenon in relation to various factors such as masculinity (36), interpersonal conflict (37), and psychosocial stressors (38). The higher proportion of female in joint suicides compared to individual suicides were also found in previous studies. However, contrary to these suicide pacts which generally involved married couples or someone with a close relationship (39–41), our study revealed that joint suicides in South Korea involved strangers whom they met for the purpose of suicide more so than their spouse.

Among all places of death, the proportion of male victims was highest in suburban or mountainous areas, followed by in school or the workplace, whereas the proportion of female victims was highest in an acquaintance's home, followed by a relative's home. Such findings coincide with the previous study in which being female significantly increased the risk of home suicide in South Korea along with a few other countries (42). The fact that female preferring home suicide more than male to be greater in the home of another compared to their own home raises questions to be further explored.

Among all suicide methods, the proportion of male victims was highest for suicide by fire, followed by suicide by transportation, and proportion of female victims was highest for suicide by drug overdose, followed by jumping from heights. Several studies have discussed the gender difference of male choosing violent or lethal methods more than female, which is a consistent trend in most countries (43–45). This has been suggested as a major contributing factor to the gender gap in completed suicides and has been attributed to reasons such as intent to die, concern for physical disfigurement and pain, inclination toward violence, and accessibility to the method (46). In terms of warning signs, men generally showed warning sign less frequently compared to women. In addition, men showed verbal signs most commonly, whereas women showed mood-related signs most commonly. This is partly related to the fact that depression, which is one of the most important causes for suicide, is much more common in female than in male (47).

### Social class and suicide

Regarding social class, first, other economically inactive individuals (typically the elderly and individuals with disabilities) represented the largest proportion of individual suicides, while employees represented the largest proportion of murder-suicides. This coincides with the finding that murder-suicide victims are significantly more likely to be employed than individual suicide victims (37), though the opposite was also observed in another study in which the employment status of both groups was similar (48). Other economically inactive individuals and employees had the two largest percentages of joint suicide. Unemployed individuals had the third largest percentage for all three suicide types. Notably, the proportion of students was higher for joint suicides than for individual suicides and murder-suicides. The relatively frequent involvement of adolescents in joint suicides supports the argument that the suicide pact victims in Eastern countries are younger than the West (41).

Second, the economically inactive group comprised the largest proportion of suicide victims for most places of death (but especially so for hospitals). Exceptions included schools or workplaces, for which self-employed individuals accounted for the largest proportion, and acquaintances' homes and accommodations, for which employees accounted for the largest proportion. Third, in terms of suicide methods, other economically inactive individuals and employees comprised the largest proportion of suicide victims for most suicide methods. One exception was pesticides, for which other economically inactive individuals accounted for the majority, followed by self-employed individuals. Moreover, the proportions of students who died by jumping from heights and drowning were higher than those for other methods.

Fourth, regarding warning signs, all social classes showed verbal and mood signs more than they showed behavioral signs. The most frequent warning signs for unemployed individuals, students, and house makers were mood-related, whereas the most frequent warning signs for other social classes were verbal. Higher rates of mood-related warning sings in house makers could be because they are mainly females. Considering low income and unemployment are important risk factors for suicide (49), suicide warning signs observed in the unemployed populations should be examined with special attention. More direct and targeted intervention should be planned. Additionally, compared to other social classes, a much higher proportion of students did not show warning signs compared to those showing any of the three warning signs. Thus, increasing suicide awareness is important for suicide prevention among students. As an example, to increase awareness of suicide, the Korea Suicide Prevention Center developed suicide prevention program for gatekeeper intervention (50). The program has focused on increasing awareness on suicide warning signs among publics including the students group.

### Occupation and suicide

In terms of occupation, first, laborers and salespeople represented the largest proportion of individual suicides. Further, the proportions of service workers and illegal business-related workers were higher for joint suicides than for other suicide types. On the other hand, proportions of mechanics and laborers were higher for murder-suicides than for other suicide types. As individuals in the service industry and illegal businesses are economically instable (35), such results contradict the previous studies in which the suicide pact victims had relatively high occupational socioeconomic status (39, 41). However, this coincides with the beforementioned gender difference in suicide types as both occupations are female oriented.

Second, in terms of place of death, the proportions of managers and salespeople who died by suicide at school or in the workplace were higher than those in other places, whereas primary industries workers and illegal business-related workers had died by suicide at higher rates in hospitals than in other places. Third, regarding suicide methods, laborers comprised the largest proportion of victims for most suicide methods. One exception was pesticides, for which primary industries workers accounted for the largest. Additionally, higher percentages of suicide for professionals were found in drug overdose and jumping from heights, while for laborers they were found in suicides by fire and wound-induced suicides. Such occupational differences in the chosen method supports the argument that an individual's choice of suicide method is influenced by how accessible and familiar it is in relation to their occupation (51, 52).

Lastly, the most frequently observed warning signs were verbal signs, followed by mood-related and behavioral signs, for all occupations except professionals, whose most frequent warning signs were mood-related. Among all occupations, primary industries workers had the highest percentages for the two verbal signs of mentioning suicide, murder, and death and mentioning physical inconvenience and discomfort, whereas illegal business-related workers displayed the highest rates of hypomania (a mood-related sign). Moreover, technicians and laborers exhibited higher rates of self-injury or substance abuse (behavioral signs) than did those in other occupations.

Overall, with the goal of linking suicide and social determinants of health, the study proposed a conceptual framework for understanding social determinants of suicide by linking a national-level suicide data in South Korea with the SDOH framework proposed by the WHO's CSDH. Within the proposed framework, by using a national-level data rather than a small sample, the study revealed strong associations between structural determinants and suicide outcomes. Yet, due to the descriptive nature of the study, we were not able to yield casual insights that will have direct implications for suicide prevention. In addition, due to the absence of studies that examined social determinants of health with comparably large-scale national suicide data, we were not able to compare our findings with others, which is important to test any generalizability.

## Conclusions

In sum, a clear connection exists between the investigated structural factors and various suicide outcomes, with gender, social class, and occupation all impacting suicide. These structural determinants operate through intermediary determinants, which we did not explore in this study. Therefore, to understand the structural determinants' mechanism more thoroughly, investigation of intermediary determinants within the present conceptual framework is necessary, which will be addressed in our follow-up study.

## Data availability statement

The original contributions presented in the study are included in the article/Supplementary material, further inquiries can be directed to the corresponding author.

## Ethics statement

The studies involving human participants were reviewed and approved by University Ethics Committee (IRB Number: SKKU 2020 01-003-001). Written informed consent for participation was not required for this study in accordance with the national legislation and the institutional requirements.

## Author contributions

YZ contributed to the conceptualization, formal analysis, investigation, methodology, and writing of the manuscript. SN contributed to the data curation, formal analysis, and writing of the manuscript. LQ contributed to the validation and writing of the manuscript. HJ and JB validated and revised the manuscript. BT revised the manuscript. All authors contributed to the article and approved the submitted version.

## Funding

This work was supported by the Ministry of Education of the Republic of Korea and the National Research Foundation of Korea (NRF-2021S1A5A8062262).

## Conflict of interest

The authors declare that the research was conducted in the absence of any commercial or financial relationships that could be construed as a potential conflict of interest.

## Publisher's note

All claims expressed in this article are solely those of the authors and do not necessarily represent those of their affiliated organizations, or those of the publisher, the editors and the reviewers. Any product that may be evaluated in this article, or claim that may be made by its manufacturer, is not guaranteed or endorsed by the publisher.

## Abbreviations

CDSH: Commission on Social Determinants of Health
SDOH: social determinants of health
WHO: World Health Organization.
