# Supplementary material for: Linking suicide and social determinants of health in South Korea: An investigation of structural determinants
Source: Front Public Health. 2022 Oct 25;10:1022790. doi: 10.3389/fpubh.2022.1022790 (PMC9641084; doi:10.3389/fpubh.2022.1022790)
Supplement: Supplementary file 1 [file Data_Sheet_1.PDF]

Supplementary Material

Supplementary Table 1. The frequency and percentage of suicide types according to social determinants (gender, social class, and occupation)

Frequency<br>% of row total<br>% of column total	Suicide type				
	Individual suicide	Joint suicide	Murder suicide	Unknown	Total
Gender					
Male	15980	183	73	262	16498
	96.86%	1.11%	0.44%	1.59%	
	69.67%	55.96%	79.35%	83.97%	
Female	6954	144	19	49	7166
	97.04%	2.01%	0.27%	0.68%	
	30.32%	44.04%	20.65%	15.71%	
Unknown	3	0	0	1	4
	75.00%	0.00%	0.00%	25.00%	
	0.01%	0.00%	0.00%	0.32%	
Total	22937	327	92	312	23668
Social class					
Employee	4820	84	32	72	5008
	96.25%	1.68%	0.64%	1.44%	
	21.01%	25.69%	34.78%	23.08%	
Self-employed	2918	23	11	35	2987
	97.69%	0.77%	0.37%	1.17%	
	12.72%	7.03%	11.96%	11.22%	
Unemployed	3139	60	14	17	3230
	97.18%	1.86%	0.43%	0.53%	
	13.69%	18.35%	15.22%	5.45%	
Student	767	24	1	14	806
	95.16%	2.98%	0.12%	1.74%	
	3.34%	7.34%	1.09%	4.49%	
Housemaker	1121	10	7	8	1146
	97.82%	0.87%	0.61%	0.70%	
	4.89%	3.06%	7.61%	2.56%	
Military/Social service worker	46	0	0	1	47
	97.87%	0.00%	0.00%	2.13%	
	0.20%	0.00%	0.00%	0.32%	
Other economically inactive	8523	79	16	104	8722
	97.72%	0.91%	0.18%	1.19%	
	37.16%	24.16%	17.39%	33.33%	
Others	1603	47	11	61	1722
	93.09%	2.73%	0.64%	3.54%	

	6.99%	14.37%	11.96%	19.55%	
Total	22937	327	92	312	23668
Occupation					
Manager	745	8	4	8	765
	97.39%	1.05%	0.52%	1.05%	
	9.38%	7.41%	9.09%	7.41%	
Professional	852	9	5	10	876
	97.26%	1.03%	0.57%	1.14%	
	10.73%	8.33%	11.36%	9.26%	
Office worker	573	9	4	4	590
	97.12%	1.53%	0.68%	0.68%	
	7.22%	8.33%	9.09%	3.70%	
Service worker	867	20	0	6	893
	97.09%	2.24%	0.00%	0.67%	
	10.92%	18.52%	0.00%	5.56%	
Salesperson	1054	11	4	11	1080
	97.59%	1.02%	0.37%	1.02%	
	13.27%	10.19%	9.09%	10.19%	
Primary industries worker	711	6	4	13	734
	96.87%	0.82%	0.54%	1.77%	
	8.95%	5.56%	9.09%	12.04%	
Technician	490	5	2	7	504
	97.22%	0.99%	0.40%	1.39%	
	6.17%	4.63%	4.55%	6.48%	
Mechanic	692	5	6	9	712
	97.19%	0.70%	0.84%	1.26%	
	8.72%	4.63%	13.64%	8.33%	
Laborer	1200	18	9	27	1254
	95.69%	1.44%	0.72%	2.15%	
	15.11%	16.67%	20.45%	25.00%	
Solider	4	0	0	0	4
	100.00%	0.00%	0.00%	0.00%	
	0.05%	0.00%	0.00%	0.00%	
Illegal business-related worker	752	17	6	13	788
	95.43%	2.16%	0.76%	1.65%	
	9.47%	15.74%	13.64%	12.04%	
Total	7940	108	44	108	8200

Supplementary Table 2. The frequency and percentage of places of death according to social determinants (gender, social class, and occupation)

Frequency<br>% of row total<br>% of column total	Place of death									
	One's own home	Home of relative	Home of acquaintance	School or workplace	Public place	Accommo dations	Suburb or mountain	Hospital	Others	Total
Gender										
Male	8193	113	81	711	4936	860	807	224	573	16498
	49.66%	0.68%	0.49%	4.31%	29.92%	5.21%	4.89%	1.36%	3.47%	
	62.56%	59.79%	55.86%	88.43%	76.88%	77.34%	92.55%	74.42%	78.60%	
Female	4903	76	64	93	1482	252	63	77	156	7166
	68.42%	1.06%	0.89%	1.30%	20.68%	3.52%	0.88%	1.07%	2.18%	
	37.44%	40.21%	44.14%	11.57%	23.08%	22.66%	7.22%	25.58%	21.40%	
Unknown	0	0	0	0	2	0	2	0	0	4
	0.00%	0.00%	0.00%	0.00%	50.00%	0.00%	50.00%	0.00%	0.00%	
	0.00%	0.00%	0.00%	0.00%	0.03%	0.00%	0.23%	0.00%	0.00%	
Total	13096	189	145	804	6420	1112	872	301	729	23668
Social class										
Employee	2465	45	49	239	1471	343	218	22	156	5008
	49.22%	0.90%	0.98%	4.77%	29.37%	6.85%	4.35%	0.44%	3.12%	
	18.82%	23.81%	33.79%	29.73%	22.91%	30.85%	25.00%	7.31%	21.40%	
Self-employed	1384	13	11	513	741	103	142	13	67	2987
	46.33%	0.44%	0.37%	17.17%	24.81%	3.45%	4.75%	0.44%	2.24%	
	10.57%	6.88%	7.59%	63.81%	11.54%	9.26%	16.28%	4.32%	9.19%	
Unemployed	1761	28	34	9	944	232	109	16	97	3230
	54.52%	0.87%	1.05%	0.28%	29.23%	7.18%	3.37%	0.50%	3.00%	
	13.45%	14.81%	23.45%	1.12%	14.70%	20.86%	12.50%	5.32%	13.31%	
Student	353	7	6	19	321	39	5	4	52	806
	43.80%	0.87%	0.74%	2.36%	39.83%	4.84%	0.62%	0.50%	6.45%	
	2.70%	3.70%	4.14%	2.36%	5.00%	3.51%	0.57%	1.33%	7.13%	
Housemaker	855	14	5	0	216	22	6	11	17	1146

	74.61%	1.22%	0.44%	0.00%	18.85%	1.92%	0.52%	0.96%	1.48%	
	6.53%	7.41%	3.45%	0.00%	3.36%	1.98%	0.69%	3.65%	2.33%	
Military/Social service worker	15	0	0	4	16	3	3	0	6	47
	31.91%	0.00%	0.00%	8.51%	34.04%	6.38%	6.38%	0.00%	12.77%	
	0.11%	0.00%	0.00%	0.50%	0.25%	0.27%	0.34%	0.00%	0.82%	
Other economically inactive	5469	67	21	12	2203	217	269	213	251	8722
	62.70%	0.77%	0.24%	0.14%	25.26%	2.49%	3.08%	2.44%	2.88%	
	41.76%	35.45%	14.48%	1.49%	34.31%	19.51%	30.85%	70.76%	34.43%	
Others	794	15	19	8	508	153	120	22	83	1722
	46.11%	0.87%	1.10%	0.46%	29.50%	8.89%	6.97%	1.28%	4.82%	
	6.06%	7.94%	13.10%	1.00%	7.91%	13.76%	13.76%	7.31%	11.39%	
Total	13096	189	145	804	6420	1112	872	301	729	23668
Occupation										
Manager	249	4	6	159	240	39	42	2	24	765
	32.55%	0.52%	0.78%	20.78%	31.37%	5.10%	5.49%	0.26%	3.14%	
	6.24%	6.90%	9.38%	21.00%	10.69%	8.46%	11.54%	5.56%	10.62%	
Professional	463	7	3	74	231	43	27	2	26	876
	52.85%	0.80%	0.34%	8.45%	26.37%	4.91%	3.08%	0.23%	2.97%	
	11.61%	12.07%	4.69%	9.78%	10.28%	9.33%	7.42%	5.56%	11.50%	
Office worker	280	3	7	30	184	44	18	2	22	590
	47.46%	0.51%	1.19%	5.08%	31.19%	7.46%	3.05%	0.34%	3.73%	
	7.02%	5.17%	10.94%	3.96%	8.19%	9.54%	4.95%	5.56%	9.73%	
Service worker	465	7	7	73	232	65	22	2	20	893
	52.07%	0.78%	0.78%	8.17%	25.98%	7.28%	2.46%	0.22%	2.24%	
	11.66%	12.07%	10.94%	9.64%	10.33%	14.10%	6.04%	5.56%	8.85%	
Salesperson	516	10	10	156	262	62	38	3	23	1080
	47.78%	0.93%	0.93%	14.44%	24.26%	5.74%	3.52%	0.28%	2.13%	
	12.94%	17.24%	15.63%	20.61%	11.67%	13.45%	10.44%	8.33%	10.18%	
Primary industries worker	431	5	2	99	120	5	47	7	18	734
	58.72%	0.68%	0.27%	13.49%	16.35%	0.68%	6.40%	0.95%	2.45%	
	10.81%	8.62%	3.13%	13.08%	5.34%	1.08%	12.91%	19.44%	7.96%	
Technician	223	4	2	58	152	33	19	3	10	504

	44.25%	0.79%	0.40%	11.51%	30.16%	6.55%	3.77%	0.60%	1.98%	
	5.59%	6.90%	3.13%	7.66%	6.77%	7.16%	5.22%	8.33%	4.42%	
Mechanic	347	5	8	39	225	27	40	5	16	712
	48.74%	0.70%	1.12%	5.48%	31.60%	3.79%	5.62%	0.70%	2.25%	
	8.70%	8.62%	12.50%	5.15%	10.02%	5.86%	10.99%	13.89%	7.08%	
Laborer	610	9	12	48	367	94	71	3	40	1254
	48.64%	0.72%	0.96%	3.83%	29.27%	7.50%	5.66%	0.24%	3.19%	
	15.30%	15.52%	18.75%	6.34%	16.34%	20.39%	19.51%	8.33%	17.70%	
Solider	1	0	0	0	2	1	0	0	0	4
	25.00%	0.00%	0.00%	0.00%	50.00%	25.00%	0.00%	0.00%	0.00%	
	0.03%	0.00%	0.00%	0.00%	0.09%	0.22%	0.00%	0.00%	0.00%	
Illegal business-related worker	403	4	7	21	231	48	40	7	27	788
	51.14%	0.51%	0.89%	2.66%	29.31%	6.09%	5.08%	0.89%	3.43%	
	10.11%	6.90%	10.94%	2.77%	10.28%	10.41%	10.99%	19.44%	11.95%	
Total	3988	58	64	757	2246	461	364	36	226	8200

Supplementary Table 3. The frequency and percentage of suicide methods according to social determinants (gender, social class, and occupation)

Frequency<br>% of row total<br>% of column total	Suicide method											
	Drug overdose	Pesticide	Carbon monoxide	Hanging	Firearm	Jumping from height	Wounding	Drowning	Transportation	Fire	Others	Total
Gender												
Male	257	1403	2659	8762	9	2083	221	932	42	91	39	16498
	1.56%	8.50%	16.12%	53.11%	0.05%	12.63%	1.34%	5.65%	0.25%	0.55%	0.24%	
	51.71%	66.68%	79.28%	71.33%	100.00%	56.50%	78.37%	75.10%	80.77%	88.35%	69.64%	
Female	240	701	695	3517	0	1604	61	309	10	12	17	7166
	3.35%	9.78%	9.70%	49.08%	0.00%	22.38%	0.85%	4.31%	0.14%	0.17%	0.24%	
	48.29%	33.32%	20.72%	28.63%	0.00%	43.50%	21.63%	24.90%	19.23%	11.65%	30.36%	
Unknown	0	0	0	4	0	0	0	0	0	0	0	4
	0.00%	0.00%	0.00%	100.00%	0.00%	0.00%	0.00%	0.00%	0.00%	0.00%	0.00%	
	0.00%	0.00%	0.00%	0.03%	0.00%	0.00%	0.00%	0.00%	0.00%	0.00%	0.00%	
Total	497	2104	3354	12283	9	3687	282	1241	52	103	56	23668
Social class												
Employee	78	207	1006	2755	3	576	64	271	9	29	10	5008
	1.56%	4.13%	20.09%	55.01%	0.06%	11.50%	1.28%	5.41%	0.18%	0.58%	0.20%	
	15.69%	9.84%	29.99%	22.43%	33.33%	15.62%	22.70%	21.84%	17.31%	28.16%	17.86%	
Self-employed	37	387	524	1722	2	205	21	71	2	13	3	2987
	1.24%	12.96%	17.54%	57.65%	0.07%	6.86%	0.70%	2.38%	0.07%	0.44%	0.10%	
	7.44%	18.39%	15.62%	14.02%	22.22%	5.56%	7.45%	5.72%	3.85%	12.62%	5.36%	
Unemployed	61	133	663	1696	3	432	43	172	8	13	6	3230
	1.89%	4.12%	20.53%	52.51%	0.09%	13.37%	1.33%	5.33%	0.25%	0.40%	0.19%	
	12.27%	6.32%	19.77%	13.81%	33.33%	11.72%	15.25%	13.86%	15.38%	12.62%	10.71%	
Student	8	9	101	241	0	328	3	114	1	1	0	806
	0.99%	1.12%	12.53%	29.90%	0.00%	40.69%	0.37%	14.14%	0.12%	0.12%	0.00%	
	1.61%	0.43%	3.01%	1.96%	0.00%	8.90%	1.06%	9.19%	1.92%	0.97%	0.00%	
Housemaker	28	69	69	649	0	276	11	39	3	1	1	1146
	2.44%	6.02%	6.02%	56.63%	0.00%	24.08%	0.96%	3.40%	0.26%	0.09%	0.09%	

	5.63%	3.28%	2.06%	5.28%	0.00%	7.49%	3.90%	3.14%	5.77%	0.97%	1.79%	
Military/Social service worker	1	0	5	18	0	11	0	11	1	0	0	47
	2.13%	0.00%	10.64%	38.30%	0.00%	23.40%	0.00%	23.40%	2.13%	0.00%	0.00%	
	0.20%	0.00%	0.15%	0.15%	0.00%	0.30%	0.00%	0.89%	1.92%	0.00%	0.00%	
Other economically inactive	231	1175	662	4329	1	1676	127	438	23	34	26	8722
	2.65%	13.47%	7.59%	49.63%	0.01%	19.22%	1.46%	5.02%	0.26%	0.39%	0.30%	
	46.48%	55.85%	19.74%	35.24%	11.11%	45.46%	45.04%	35.29%	44.23%	33.01%	46.43%	
Others	53	124	324	873	0	183	13	125	5	12	10	1722
	3.08%	7.20%	18.82%	50.70%	0.00%	10.63%	0.75%	7.26%	0.29%	0.70%	0.58%	
	10.66%	5.89%	9.66%	7.11%	0.00%	4.96%	4.61%	10.07%	9.62%	11.65%	17.86%	
Total	497	2104	3354	12283	9	3687	282	1241	52	103	56	23668
Occupation												
Manager	7	15	166	455	1	79	8	31	1	2	0	765
	0.92%	1.96%	21.70%	59.48%	0.13%	10.33%	1.05%	4.05%	0.13%	0.26%	0.00%	
	5.93%	2.48%	10.67%	9.86%	20.00%	9.89%	9.41%	8.96%	9.09%	4.55%	0.00%	
Professional	21	31	154	474	0	135	11	41	3	5	1	876
	2.40%	3.54%	17.58%	54.11%	0.00%	15.41%	1.26%	4.68%	0.34%	0.57%	0.11%	
	17.80%	5.12%	9.90%	10.27%	0.00%	16.90%	12.94%	11.85%	27.27%	11.36%	7.14%	
Office worker	5	17	134	311	0	79	2	39	1	2	0	590
	0.85%	2.88%	22.71%	52.71%	0.00%	13.39%	0.34%	6.61%	0.17%	0.34%	0.00%	
	4.24%	2.81%	8.61%	6.74%	0.00%	9.89%	2.35%	11.27%	9.09%	4.55%	0.00%	
Service worker	17	35	148	525	3	115	7	37	2	3	1	893
	1.90%	3.92%	16.57%	58.79%	0.34%	12.88%	0.78%	4.14%	0.22%	0.34%	0.11%	
	14.41%	5.78%	9.51%	11.37%	60.00%	14.39%	8.24%	10.69%	18.18%	6.82%	7.14%	
Salesperson	14	37	227	654	1	100	8	33	0	5	1	1080
	1.30%	3.43%	21.02%	60.56%	0.09%	9.26%	0.74%	3.06%	0.00%	0.46%	0.09%	
	11.86%	6.11%	14.59%	14.17%	20.00%	12.52%	9.41%	9.54%	0.00%	11.36%	7.14%	
Primary industries worker	5	309	37	357	0	11	3	8	0	2	2	734
	0.68%	42.10%	5.04%	48.64%	0.00%	1.50%	0.41%	1.09%	0.00%	0.27%	0.27%	
	4.24%	50.99%	2.38%	7.73%	0.00%	1.38%	3.53%	2.31%	0.00%	4.55%	14.29%	
Technician	6	28	100	309	0	36	6	15	0	3	1	504
	1.19%	5.56%	19.84%	61.31%	0.00%	7.14%	1.19%	2.98%	0.00%	0.60%	0.20%	

	5.08%	4.62%	6.43%	6.69%	0.00%	4.51%	7.06%	4.34%	0.00%	6.82%	7.14%	
Mechanic	11	32	179	407	0	36	7	33	0	6	1	712
	1.54%	4.49%	25.14%	57.16%	0.00%	5.06%	0.98%	4.63%	0.00%	0.84%	0.14%	
	9.32%	5.28%	11.50%	8.82%	0.00%	4.51%	8.24%	9.54%	0.00%	13.64%	7.14%	
Laborer	21	79	228	688	0	115	29	69	3	16	6	1254
	1.67%	6.30%	18.18%	54.86%	0.00%	9.17%	2.31%	5.50%	0.24%	1.28%	0.48%	
	17.80%	13.04%	14.65%	14.90%	0.00%	14.39%	34.12%	19.94%	27.27%	36.36%	42.86%	
Solider	0	0	0	1	0	2	0	1	0	0	0	4
	0.00%	0.00%	0.00%	25.00%	0.00%	50.00%	0.00%	25.00%	0.00%	0.00%	0.00%	
	0.00%	0.00%	0.00%	0.02%	0.00%	0.25%	0.00%	0.29%	0.00%	0.00%	0.00%	
Illegal business-related worker	11	23	183	435	0	91	4	39	1	0	1	788
	1.40%	2.92%	23.22%	55.20%	0.00%	11.55%	0.51%	4.95%	0.13%	0.00%	0.13%	
	9.32%	3.80%	11.76%	9.42%	0.00%	11.39%	4.71%	11.27%	9.09%	0.00%	7.14%	
Total	118	606	1556	4616	5	799	85	346	11	44	14	8200

Supplementary Table 4. The frequency and percentage of warning signs according to social determinants (gender, social class, and occupation)

Frequency<br>% of row total (distinct)<br>% of column total	Warning sign															
	Verbal				Behavioral				Mood				Unknown change	No change	Unknown	Total (distinct)
	talking about suicide, murder, and death	talking about physical inconvenience and discomfort	putting oneself down	Others	apathy to appearance	self-injury or substance abuse	lethargy, social phobia, loss of interest	Others	depression	hypomania	anxiety	Others				
Gender																
Male	7892	3214	1382	5089	103	4196	3010	3742	10247	399	2740	377	67	751	1180	16498
	47.84%	19.48%	8.38%	30.85%	0.62%	25.43%	18.24%	22.68%	62.11%	2.42%	16.61%	2.29%	0.41%	4.55%	7.15%	
	66.77%	64.69%	64.79%	70.72%	59.88%	70.76%	67.98%	67.06%	65.26%	61.01%	64.64%	61.80%	75.28%	75.86%	82.34%	
Female	3928	1754	751	2107	69	1734	1418	1838	5454	255	1499	233	22	239	249	7166
	54.81%	24.48%	10.48%	29.40%	0.96%	24.20%	19.79%	25.65%	76.11%	3.56%	20.92%	3.25%	0.31%	3.34%	3.47%	
	33.23%	35.31%	35.21%	29.28%	40.12%	29.24%	32.02%	32.94%	34.74%	38.99%	35.36%	38.20%	24.72%	24.14%	17.38%	
Unknown	0	0	0	0	0	0	0	0	0	0	0	0	0	0	4	4
	0.00%	0.00%	0.00%	0.00%	0.00%	0.00%	0.00%	0.00%	0.00%	0.00%	0.00%	0.00%	0.00%	0.00%	100.00%	
	0.00%	0.00%	0.00%	0.00%	0.00%	0.00%	0.00%	0.00%	0.00%	0.00%	0.00%	0.00%	0.00%	0.00%	0.28%	
Total	11820	4968	2133	7196	172	5930	4428	5580	15701	654	4239	610	89	990	1433	23668
Social class																
Employee	2119	581	406	1825	17	1282	741	1174	3147	134	997	138	28	268	321	5008
	42.31%	11.60%	8.11%	36.44%	0.34%	25.60%	14.80%	23.44%	62.84%	2.68%	19.91%	2.76%	0.56%	5.35%	6.41%	
	17.93%	11.69%	19.03%	25.36%	9.88%	21.62%	16.73%	21.04%	20.04%	20.49%	23.52%	22.62%	31.46%	27.07%	22.40%	
Self-employed	1480	412	222	993	17	816	433	666	1940	77	518	88	11	125	154	2987
	49.55%	13.79%	7.43%	33.24%	0.57%	27.32%	14.50%	22.30%	64.95%	2.58%	17.34%	2.95%	0.37%	4.18%	5.16%	
	12.52%	8.29%	10.41%	13.80%	9.88%	13.76%	9.78%	11.94%	12.36%	11.77%	12.22%	14.43%	12.36%	12.63%	10.75%	

Unemployed	1439	413	301	1125	33	952	722	866	2248	73	633	77	17	116	191	3230
	44.55%	12.79%	9.32%	34.83%	1.02%	29.47%	22.35%	26.81%	69.60%	2.26%	19.60%	2.38%	0.53%	3.59%	5.91%	
	12.17%	8.31%	14.11%	15.63%	19.19%	16.05%	16.31%	15.52%	14.32%	11.16%	14.93%	12.62%	19.10%	11.72%	13.33%	
Student	296	60	74	277	6	113	169	203	475	27	170	31	4	78	49	806
	36.72%	7.44%	9.18%	34.37%	0.74%	14.02%	20.97%	25.19%	58.93%	3.35%	21.09%	3.85%	0.50%	9.68%	6.08%	
	2.50%	1.21%	3.47%	3.85%	3.49%	1.91%	3.82%	3.64%	3.03%	4.13%	4.01%	5.08%	4.49%	7.88%	3.42%	
Housemaker	620	315	109	343	10	290	252	330	951	44	283	54	4	41	23	1146
	54.10%	27.49%	9.51%	29.93%	0.87%	25.31%	21.99%	28.80%	82.98%	3.84%	24.69%	4.71%	0.35%	3.58%	2.01%	
	5.25%	6.34%	5.11%	4.77%	5.81%	4.89%	5.69%	5.91%	6.06%	6.73%	6.68%	8.85%	4.49%	4.14%	1.61%	
Military/Social service worker	23	5	5	16	1	7	13	15	30	4	11	1	1	0	4	47
	48.94%	10.64%	10.64%	34.04%	2.13%	14.89%	27.66%	31.91%	63.83%	8.51%	23.40%	2.13%	2.13%	0.00%	8.51%	
	0.19%	0.10%	0.23%	0.22%	0.58%	0.12%	0.29%	0.27%	0.19%	0.61%	0.26%	0.16%	1.12%	0.00%	0.28%	
Other economically inactive	5150	2964	920	2072	82	2070	1919	1893	6019	246	1386	168	19	291	352	8722
	59.05%	33.98%	10.55%	23.76%	0.94%	23.73%	22.00%	21.70%	69.01%	2.82%	15.89%	1.93%	0.22%	3.34%	4.04%	
	43.57%	59.66%	43.13%	28.79%	47.67%	34.91%	43.34%	33.92%	38.34%	37.61%	32.70%	27.54%	21.35%	29.39%	24.56%	
Others	693	218	96	545	6	400	179	433	891	49	241	53	5	71	339	1722
	40.24%	12.66%	5.57%	31.65%	0.35%	23.23%	10.39%	25.15%	51.74%	2.85%	14.00%	3.08%	0.29%	4.12%	19.69%	
	5.86%	4.39%	4.50%	7.57%	3.49%	6.75%	4.04%	7.76%	5.67%	7.49%	5.69%	8.69%	5.62%	7.17%	23.66%	
Total	11820	4968	2133	7196	172	5930	4428	5580	15701	654	4239	610	89	990	1433	23668
Occupation																
Manager	332	85	74	298	5	168	117	194	485	11	182	28	2	37	28	765
	43.40%	11.11%	9.67%	38.95%	0.65%	21.96%	15.29%	25.36%	63.40%	1.44%	23.79%	3.66%	0.26%	4.84%	3.66%	
	8.96%	8.33%	11.42%	10.24%	14.71%	7.78%	9.79%	10.08%	9.26%	5.02%	11.58%	11.81%	5.13%	9.20%	5.81%	
Professional	358	112	78	348	2	170	142	240	596	22	218	29	6	41	47	876
	40.87%	12.79%	8.90%	39.73%	0.23%	19.41%	16.21%	27.40%	68.04%	2.51%	24.89%	3.31%	0.68%	4.68%	5.37%	
	9.66%	10.98%	12.04%	11.96%	5.88%	7.87%	11.88%	12.47%	11.37%	10.05%	13.88%	12.24%	15.38%	10.20%	9.75%	
Office	223	62	61	245	3	105	98	163	383	13	146	14	2	28	36	590

worker	37.80%	10.51%	10.34%	41.53%	0.51%	17.80%	16.61%	27.63%	64.92%	2.20%	24.75%	2.37%	0.34%	4.75%	6.10%	
	6.02%	6.08%	9.41%	8.42%	8.82%	4.86%	8.20%	8.47%	7.31%	5.94%	9.29%	5.91%	5.13%	6.97%	7.47%	
Service worker	416	103	67	284	4	252	102	172	585	24	152	21	4	43	51	893
	46.58%	11.53%	7.50%	31.80%	0.45%	28.22%	11.42%	19.26%	65.51%	2.69%	17.02%	2.35%	0.45%	4.82%	5.71%	
	11.23%	10.10%	10.34%	9.76%	11.76%	11.67%	8.54%	8.94%	11.16%	10.96%	9.68%	8.86%	10.26%	10.70%	10.58%	
Salesperson	512	119	103	397	6	287	169	276	725	28	238	46	6	52	50	1080
	47.41%	11.02%	9.54%	36.76%	0.56%	26.57%	15.65%	25.56%	67.13%	2.59%	22.04%	4.26%	0.56%	4.81%	4.63%	
	13.82%	11.67%	15.90%	13.64%	17.65%	13.29%	14.14%	14.35%	13.84%	12.79%	15.15%	19.41%	15.38%	12.94%	10.37%	
Primary industries worker	392	160	30	200	6	209	113	142	471	24	79	10	4	35	25	734
	53.41%	21.80%	4.09%	27.25%	0.82%	28.47%	15.40%	19.35%	64.17%	3.27%	10.76%	1.36%	0.54%	4.77%	3.41%	
	10.58%	15.69%	4.63%	6.87%	17.65%	9.68%	9.46%	7.38%	8.99%	10.96%	5.03%	4.22%	10.26%	8.71%	5.19%	
Technician	221	65	32	155	0	160	71	111	316	9	77	12	4	30	27	504
	43.85%	12.90%	6.35%	30.75%	0.00%	31.75%	14.09%	22.02%	62.70%	1.79%	15.28%	2.38%	0.79%	5.95%	5.36%	
	5.96%	6.37%	4.94%	5.33%	0.00%	7.41%	5.94%	5.77%	6.03%	4.11%	4.90%	5.06%	10.26%	7.46%	5.60%	
Mechanic	340	79	47	236	3	205	94	151	428	18	132	23	2	38	55	712
	47.75%	11.10%	6.60%	33.15%	0.42%	28.79%	13.20%	21.21%	60.11%	2.53%	18.54%	3.23%	0.28%	5.34%	7.72%	
	9.17%	7.75%	7.25%	8.11%	8.82%	9.49%	7.87%	7.85%	8.17%	8.22%	8.40%	9.70%	5.13%	9.45%	11.41%	
Laborer	558	164	87	428	3	394	181	252	758	26	186	30	7	51	121	1254
	44.50%	13.08%	6.94%	34.13%	0.24%	31.42%	14.43%	20.10%	60.45%	2.07%	14.83%	2.39%	0.56%	4.07%	9.65%	
	15.06%	16.08%	13.43%	14.71%	8.82%	18.24%	15.15%	13.10%	14.47%	11.87%	11.84%	12.66%	17.95%	12.69%	25.10%	
Solider	3	2	2	0	0	0	2	0	2	1	0	0	0	0	1	4
	75.00%	50.00%	50.00%	0.00%	0.00%	0.00%	50.00%	0.00%	50.00%	25.00%	0.00%	0.00%	0.00%	0.00%	25.00%	
	0.08%	0.20%	0.31%	0.00%	0.00%	0.00%	0.17%	0.00%	0.04%	0.46%	0.00%	0.00%	0.00%	0.00%	0.21%	
Illegal business-related worker	351	69	67	319	2	210	106	223	491	43	161	24	2	47	41	788
	44.54%	8.76%	8.50%	40.48%	0.25%	26.65%	13.45%	28.30%	62.31%	5.46%	20.43%	3.05%	0.25%	5.96%	5.20%	
	9.47%	6.76%	10.34%	10.96%	5.88%	9.72%	8.87%	11.59%	9.37%	19.63%	10.25%	10.13%	5.13%	11.69%	8.51%	
Total	3706	1020	648	2910	34	2160	1195	1924	5240	219	1571	237	39	402	482	8200
